# Development of an Evidence-based Nutritional Intervention Protocol for Adolescent Athletes 

**DOI:** 10.20463/jenb.2019.0020

**Published:** 2019-09-30

**Authors:** Saningun Lee, Hyunjung Lim

**Affiliations:** 1. Department of Medical Nutrition, Kyung Hee University, Yongin Republic of Korea; 2. Research Institute of Medical Nutrition, Kyung Hee University, Seoul Republic of Korea

**Keywords:** Sports nutrition, adolescent athletes, 5 A’s counseling, nutrition intervention, behavior change, motivational interviewing

## Abstract

**[Purpose]:**

Effective nutritional intervention can help reduce the risk of nutritional problems and improve athletic performance in adolescents. The five A’s (assess, advise, agree, assist, and arrange) model is widely used as the theoretical framework for advice on nutrition, smoking, drinking, and physical activity and it recommends that practitioners in primary health care promote behavior change to facilitate positive outcomes. This model has also been useful in understanding the underlying processes of behavior change. This study aimed to develop both a novel evidence-based nutritional intervention protocol, rooted in sound nutritional theory, and a customizable nutritional intervention program to support sustainable healthy eating, enhance nutrient intake, and improve athletic performance in adolescent athletes.

**[Methods]:**

In this study, we adapted the 5 A’s behavioral change model and motivational interview to develop a theoretical framework to help adolescent athletes change their behavior and achieve their goals.

**[Results]:**

During each step of the 5 A’s protocol, a customized nutritional intervention protocol was developed by nutrition experts for each of adolescent athletes. Each plan was developed to improve the eating habits of adolescent athletes through group education and counseling. All nutritional counseling sessions were designed to enable participants to apply nutritional knowledge and practical action plans to their training and competition conditions to enable each of them to achieve individual athletic goals and facilitate self-management.

**[Conclusion]:**

A theoretical and evidence-based nutritional intervention protocol was developed to identify and address obstacles to healthy dietary habits in adolescent athletes. This could be used as the basis for further studies aimed at improving nutrient intake and athletic performance in adolescent athletes.

## INTRODUCTION

An individual’s lifelong dietary and physical activity habits can be determined by his or her lifestyle during adolescence^1^. Undergoing a period of nutritional risk marked by profound psychological, physiological, and social changes^2^, adolescents need adequate nutrition to support both rapid growth and fulfillment of their physiological potential^3^. Unfortunately, adolescent athletes do not always choose healthy foods^4^ or have adequate knowledge about nutrition to allow them to select the best food to support them during competition^5^. It has been reported that, despite knowing the importance of nutrition in athletic performance, many athletes do not follow diets that meet the standards for good sports nutrition or basic health^6^. In addition, research into intervention programs focused on changing nutrition-related behaviors among adolescent athletes is limited^5^. Therefore, it is essential to understand how to design and implement appropriate and accessible behavioral programs to address and improve nutrition-related issues in adolescent athletes^7^.

Studies based on behavioral change theories including the transtheoretical model^8^^-^^11^ have confirmed the effects of successful nutritional interventions. In particular, the 5 A's (assess, advise, agree, assist, and arrange) behavioral change model^12^ has been widely used as a theoretical framework for advice on nutrition, smoking, drinking and physical activity. It has also proven to be a useful method for understanding the processes underlying behavioral change^13^. In addition, motivational interviewing (MI), a client-centric approach, to encourage intrinsic motivation^14^ and change through ambivalence^15^, is considered an effective evidence-based behavioral change strategy for adults and teenagers. In this context, nutritional education through the application of the 5 A's behavioral change counseling model with the addition of MI can be effective in improving dietary habits and nutrient intake in adolescent athletes.

Adolescent athletes who undergo high-intensity training require unique energy and nutritional intake^1^. Thus, the diet of these athletes needs to meet increased energy and nutritional demands associated with both general physical activity and specific high intensity training needs, while still reflecting individual food preferences^16^^,^^17^. Despite having higher nutritional needs, adolescent athletes are vulnerable to dietary risks, including skipped meals, fad diets, eating disorders, and marketing claims for sports supplements that promise improved performance^18^. Effective nutritional education and counseling can help adolescent athletes choose varied nutrition-dense foods, help them to maintain proper hydration, and eat balanced meals and snacks^19^.

Therefore, this study aimed to develop a theory- and evidence-based nutritional intervention program which supports sustainable healthy eating habits, enhanced nutrient intake, and improved athletic performance in these adolescent athletes.

## METHODS

### Pilot study

In the pilot study, the nutrition-related problems of adolescent athletes were segregated by the type of sport and then evaluated. These data were then used to develop a nutritional intervention protocol. Regardless of the type of sport, the pilot study found the following nutritional problems:

1. Daily energy intake was insufficient when compared with the sports nutrition recommendation (SNR) guidelines.
2. Carbohydrate intake was insufficient when compared with the SNR.
3. Intake of vitamins C, A, and D was insufficient when compared with the dietary reference intake for Koreans 2015 (KDRI).
4. Consumption of calcium and potassium was inadequate when compared with the KDRI.
5. Knowledge of nutritional concepts was low among adolescent athletes.
6. Many adolescent athletes were skipping breakfast.
7. Adolescent athletes were consuming insufficient fruits and vegetables.
8. Adolescent athletes were consuming insufficient milk and dairy products.
9. Many adolescent athletes attempted fasting to lose weight.
10. Adolescent athletes were interested in quicker recovery, reductions in body fat, and muscle gain.

A nutritional intervention protocol was developed to improve these nutritional issues in adolescent athletes.

### Literature review

Research papers from 2008 to 2018 were reviewed by a trained clinical dietitian to develop an evidence-based nutritional intervention program for adolescent athletes. Systematic literature searches of Google Scholar and MEDLINE® using the terms nutrition & athletic performance, as well as athlete & nutrition-related factors (nutrient intake, dietary habits, nutrition knowledge, nutritional considerations), adolescent athletes, nutrition education & counseling, nutrition intervention, study protocol, behavioral change, self-management, motivational interviewing, and 5 A’s model were conducted. The components of effective interventions were then reviewed and their efficacy in previous nutritional intervention studies analyzed.

### Theoretical background

**Figure 1.**
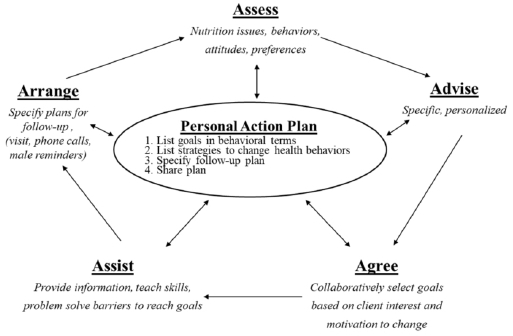
Glasgow et al.’s 5 A’s model of self-management support

This protocol was designed using the 5 A's behavioral change model (assess, advise, agree, assist, and arrange)^20^ and motivational interviewing (MI)^21^. The 5 A's framework has become a universal approach to encouraging behavioral change, including in smoking, nutrition, alcohol, and physical activity^13^. Figure 1 shows the five steps of the 5 A's model^22^: 1) Assess the subject's beliefs, behavior, and knowledge; 2) Advise the subject by providing specific information about health risks and the benefits of change; 3) Agree to collaboratively set goals based on the subject's interest and confidence in their ability to change their behavior; 4) Assist in identifying personal barriers, strategies, problem-solving techniques, and social/environmental support; 5) Arrange a plan for follow-up, such as visits, phone calls, and mailed reminders.

**Figure 2.**
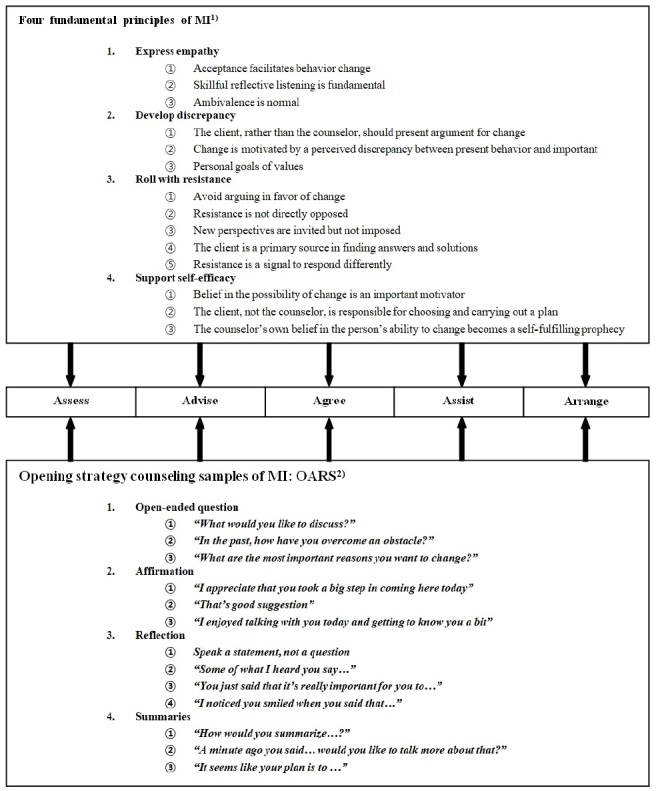
The basic principles and opening strategies of MI applied to the 5 A’s behavioral change model ^1)^ MI: motivational interviewing^2)^ OARS: open-ended questions, affirmations, reflections, and summaries

Motivating change is essential in guiding and maintaining goal-related behaviors^23^. MI is a technique for exploring and resolving ambivalence about one's health behaviors in favor of a change (24). The four fundamental principles of MI help subjects change their behavior: 1) Express empathy; 2) Develop discrepancy; 3) Roll with resistance; 4) Support self-efficacy^15^. MI can be enhanced by open-ended questions, affirmations, reflections, and summaries (OARS). Figure 2 summarizes the basic principles of MI and OARS and explains how these work within the 5 A’s model.

The nutritional intervention protocol used in this study combined the 5 A's behavioral change model with MI to improve the dietary habits and athletic performance of adolescent athletes. The combination of MI and OARS principles facilitated positive behavior modifications among adolescent athletes during each counseling step of the 5 A's model.

### Study design, setting and subjects

This protocol could be tested using either randomized experimental or quasi-experimental designs. This protocol was developed to assess the effects of nutritional interventions using the 5 A's model to address dietary problems in adolescent athletes. This protocol was also developed for application in physical education programs in Korean middle/high schools. This protocol was recommended to adolescent athletes who were training for various sporting competitions. All the data was generated from individuals who agreed to participate in the study, and voluntarily signed the written consent.

### Intervention scheme

**Figure 3.**
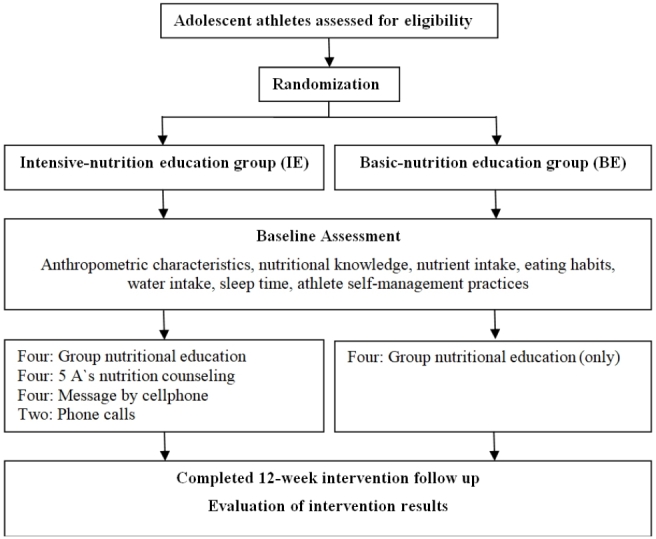
Intervention flowchart for the study

This study developed a nutritional counseling program based on the 5 A's framework. The counseling program consisted of four 20/ 30-minute sessions. The 5 A's framework has the potential to improve the efficacy of nutritional counseling by gaining participants’ consent to work toward common nutritional management goals and using these goals to improve their overall health and athletic performance. One of the priorities of the 5 A's counseling in this study was the increase of healthy eating habits in participants, with little to no focus on the prohibition of inappropriate eating habits. The study subjects were randomly assigned to the intensive nutritional education group (IE group) or the basic nutritional education group (BE group). A flowchart of the overall intervention plans in this study are presented in Figure 3. Anthropometric characteristics, namely, nutritional knowledge, nutrient intake, eating habits, water intake, sleep time, and the athletic self-management practices of the subjects were established at the beginning (baseline) and end (12-week follow-up) of the study.

## RESULTS

### Nutritional intervention protocol and program development

**Figure 4.**
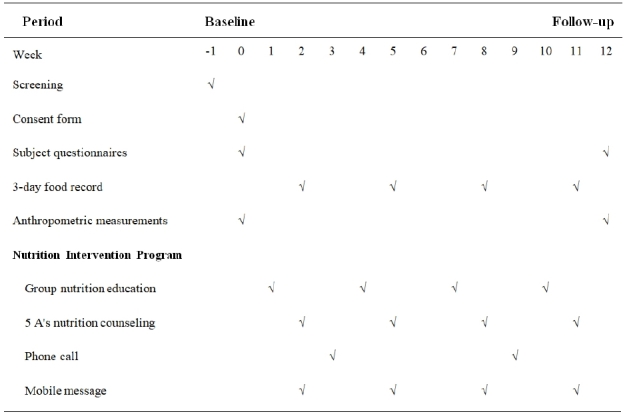
Nutritional intervention protocol

The 12-week nutritional intervention protocol is presented in Figure 4. For the baseline assessments, the subjects' consent forms, questionnaires, and 3-day food records were collected by a trained clinical dietitian. Anthropometric measurements (height, weight, body mass index, lean body mass, and body fat) were established at the beginning and end of the study. The nutritional intervention program included group education, 5 A's counseling, phone calls, and mobile messages.

### Components of the nutritional education group sessions

Nutritional experts designed a curriculum focused on four topics with 16 content areas important in sports nutrition, details of the program are in Table 1. This curriculum used ‘The Performance Nutrition Curriculum’ developed in a previous study as a base as it is suitable for and easily understood by adolescent athletes^25^. These group education sessions allowed the adolescent athletes to 1) understand the concept of sports nutrition, 2) apply that nutritional knowledge to training and competitions, and 3) eat healthily and efficiently for training and competition. During the 12 weeks of the nutritional intervention program a total of four 40-minute education sessions were conducted.

**Table 1.** Components of the four group nutritional education sessions

Session	Topic	Contents	Period
1	Basic nutrition concepts	1) How to use nutrition information 2) Eat at regular intervals 3) Maintain adequate hydration 4) Have a healthy body image	40 minutes
2	Basic food skills	1) Plan meals and shop for groceries 2) Develop basic cooking skills 3) Increase the nutrient content of meals 4) Handle and store food safely	40 minutes
3	Performance nutrition	1) Fuel before practice or competition 2) Fuel during practice or competition 3) Fuel for recovery 4) Eat for performance while traveling	40 minutes
4	Performance enhancement	1) Optimize body weight and body composition 2) Continue to gradually improve eating habits 3) Use supplements safely 4) Adjust nutrition for off-season	40 minutes

The first session, ‘Basic nutritional concepts,’ provided nutritional information like the importance of eating food at regular intervals, maintaining adequate hydration, and having a healthy body image. In the second session participants learnt about ‘Basic food skills’. This session was designed to enable adolescent athletes to plan meals, develop basic cooking skills, increase the nutrient content of their meals, and handle food safely. In the third session, the adolescent athletes learned about ‘Performance nutrition; the core of sports nutrition,’ which explains optimal food intake before, during, and after exercise and while traveling. The last session, 'Performance enhancement,' discussed how to optimize body composition, gradually improve eating habits, and use supplements safely for athletic performance (Table 1).

### Nutritional counseling scheme using a modified 5 A’s method for adolescent athletes

An evidence-based nutritional intervention protocol was developed using the 5 A's model for adolescent athletes. The intervention program was designed for implementation over a 12-week period. Each 5 A’s nutritional counseling session used all five steps: assess, advise, agree, assist, and arrange. Each participant attended four 20/30-minute nutritional counseling sessions conducted by a nutrition expert, who encouraged the subjects between sessions using cell phone messages and phone calls. Nutritional counseling was personalized to the athletic goals of each subject. Nutrition counseling was performed following an initial analysis of the dietary and exercise habits, with the goal of improving the quality of the meals adolescent athletes were eating. MI skills, which promote subject-centered behavioral changes, were applied at all stages of this program.

#### Step 1: Assess

To assess the nutritional status of the subjects, in the first step of this model (assess), dietary habits, nutrient intake, nutritional knowledge, sports supplement intake, lifestyle, appetite, health history, and anthropometrics were established by a nutrition expert. Before starting the full-scale 5 A's counseling, subjects were asked for permission using questions like "Are you concerned about the effects of your diet on your athletic performance?" and "Would it be alright if we discussed your weight-class?". In this “assess” step, it is essential to form co-operative relationships with the subjects to ensure a smooth transition to the next stage. Table 2 summarizes the role that a nutritional expert should play in this first step of the process. At this step, nonjudgmental questions, posed using motivational interviewing skills, are essential for effective communication between participants as well as generating the necessary co-operative landscape. It should be noted that questions in this step should be largely open, to encourage participants to think more about the answers.

**Table 2.** Assess step of the 5 A’s

Definition	Rationale	Counseling sample	MI^1)^
Ask permission to discuss nutrition-related factors with the subject Ask nonjudgmental questions Discuss readiness for change	Vocabulary such as body weight, body fat, and muscle mass, could be a sensitive issue Avoid linguistic clues suggesting judgment An indication of readiness might make the results predictable	*“May I talk with* *you about * *nutrition-related* *factors for your* *athletic * *performance?”* ** *“How do you* *think nutrient intake* *is important* *to your training?”*	Ask permission Explore readiness to change; use nonjudgmental questions
Assess nutrition knowledge, dietary habits, 3-day record, and anthropometry^2)^ Explore causes of inadequate nutrient intake by the subject	Body fat percentage alone should not be used as an index for nutrition management Nutrition management of athletes is complex and heterogeneous Nutrition-related problems for athletes vary from person to person.	*"What do you* *think about your* *dietary habits?"* ** ** ** ** ** ** ** ** ** ** ** ** *“What effort* *do you have to* *make to fit into* *your weight-class* *for * *a competition?”*	Open-ended questions Open-ended questions

^1)^ motivational interviewing

^2)^ weight, height, body mass index, body fat, muscle mass

#### Step 2: Advise

In the ‘advise’ step, problem behaviors identified in the assess step were reported back to the subject. He or she then learned about the importance and benefits of behavioral change in improving their athletic performance, as well as the risks associated with poor nutrition. The nutritional expert asked the subjects for permission to give them advice, including a nutritional management plan to increase the probability of behavioral change (i.e., “Now I understand your situation better, may I recommend a nutrition plan for before/during/after training to improve your endurance?”). Subjects tended to be more open to change when the expert expressed empathy, another principle of MI, during the advice step. Table 3 explains the ‘advise’ step of the 5 A's. 

**Table 3.** Advise step of the 5 A’s

Definition	Rationale	Counseling sample	MI^1)^
Advise on 1) decreased exercise performance caused by inadequate nutrition 2) benefits of optimal nutrient intake 3) the need for a long-term strategy	The recommendations for calories and nutrients vary by sport Maintaining optimal physical conditioning through proper nutrition can also benefit health and exercise performance Clear, specific and personalized nutritional advice is needed	*“Now, I understood* *your situation* *better. May* *I recommend a* *nutrition plan for* *before/during/* *after training to* *improve your* *endurance?”* *"I know how* *difficult it is to* *maintain your* *weight for a* *competition"* *"It is natural to* *have no appetite* *in the morning.* *The method I* *recommend is ..."*	Ask permission Affirmation Express empathy

^1)^ motivational interviewing

#### Step 3: Agree

In the ‘agree’ step, the subject worked collaboratively with the nutritional expert to establish nutrition goals based on their problem behaviors, interests, and confidence in successfully employing their action plan. The ‘advise’ step consisted of a respectful negotiation process in which the protagonist was not the expert but rather the subject. To address the nutrition-related problems identified in Korean adolescent during the pilot study, 10 healthy eating habits were developed for the nutritional experts to provide to the study subjects: 1) Eat food evenly at every meal; 2) Eat enough for breakfast; 3) Consume milk or dairy products 2–3 times a day; 4) Eat enough protein and iron-rich foods; 5) Eat 2–3 snacks in addition to three meals a day; 6) Drink water before, during, and after training; 7) Consume carbohydrates before exercise; 8) Eat food within 30 minutes after high-intensity training to allow for recovery; 9) Get enough sleep; 10) Choose an appropriate sports supplement (Table 4). At each nutritional counseling session, the nutritional expert emphasized those 10 healthy eating habits. To help each subject develop an action plan, such as "I'll eat protein powder and white bread for quick recovery right after exercise," the expert used practical behavior modification principles, including goal setting and behavioral shaping. Goal setting was easily accomplished using the SMART framework (specific, measurable, achievable, rewarding, and timely)^26^. The action plans built during the 'agree' step are presented in Table 5. The MI discrepancy rules helped experts and athletes set goals collaboratively. 

**Table 4.** 10 healthy eating habits for Korean adolescent athletes

No	Eating habits for athletes
1	Eat food evenly at every meal
2	Eat enough for breakfast
3	Eat milk or dairy products 2–3 times a day
4	Eat enough protein and iron-rich food
5	Eat 2–3 snacks, in addition to three meals a day
6	Drink water before, during, and after exercise
7	Eat carbohydrates before exercise
8	Eat food for recovery within 30 minutes of completing a high-intensity workout
9	Get enough sleep
10	Choose an appropriate sports supplement

**Table 5.** Agree step of the 5 A’s

Definition	Rationale	Counseling sample	MI^1)^
Agree on 1) optimal body composition and nutrient intake strategy for athletic performance improvement. 2) behavioral changes using the SMART^2)^ framework 3) specific nutritional strategies for before/during/ after training	Nutrition counseling should focus on practical behavior changes by the athlete Collaboratively set nutrition goals based on the athlete's interest and confidence that they can perform the behavior	*“On a scale* *of 1-10, how* *important is it for* *you to increase* *your muscle for* *competition?”* ** ** ** ** *“As an elite athlete,* *let me know* *the goals you* *would like to set* *up to fulfill your* *dreams”* ** *“Why would you* *want to make* *this change?”*	Discrepancy ruler Open-ended question Affirmation Desire

^1)^ motivational interviewing

^2)^ SMART; specific, measurable, achievable, rewarding, timely

#### Step 4: Assist

After agreeing on an action plan, the expert provided each participant with problem-solving strategies during the ‘assist’ step. This step used the following approach: 1) identify personal barriers; 2) manage a busy training schedule; 3) plan meals and snacks; 4) eat out in a healthy way; 5) plan for travel and restaurant meals during competition seasons; 6) make healthy food choices; 7) recommend sports supplements, if needed. The expert assisted subjects as they sought out reliable sources of nutritional information and established an appropriate support network including personal trainers and coaches. The subjects' action plans were also reviewed by the nutritional expert. Table 6 illustrates the strategy during the “assist” step.

**Table 6.** Assist step of the 5 A’s

Definition	Rationale	Counseling sample	MI^1)^
Assist in 1) identifying and addressing barriers 2) providing problem-solving strategies and resources 3) reviewing the action plan	Most athletes have substantial barriers to nutrition management, including busy training schedules and a variety of competitions Athletes might find it difficult to discern credible and non-credible sources of nutritional information	*“If you did decide* *to eat protein* *and iron-rich* *food, what do* *you think that* *would look like?”* ** ** ** *“What is the most* *difficult thing* *about achieving* *this goal?”*	Open-ended question Commitment Open-ended question Affirmation

^1)^ motivational interviewing

#### Step 5: Arrange

The 'arrange' step is important because it enabled the subjects to continue their behavioral change even after ending the formal program. Therefore, the expert provided ongoing assistance by scheduling follow-up contacts in person, by phone, phone message, and email. At the end of counseling, the expert provided group nutritional education notices and materials including various leaflets to the subjects. Table 7 demonstrates the role of the nutritional expert during the 'arrange' step.

**Table 7.** Arrange step of the 5 A’s

Definition	Rationale	Counseling sample	MI^1)^
Schedule follow-up contracts to provide ongoing assistance 1) in person 2) by cell phone 3) by phone message 4) by email	The 'Arrange’ step is important for behavioral change because it provides an opportunity 1) for follow-up 2) to reassess one’s behavioral change 3) to adjust the action plan	*“Please send me* *a message next* *week about how* *the action plan* *for increasing * *fluid intake* *during exercise* *is going. How* *confident are you* *that you can do it* *well?”* ** *“To increase your* *nutrition knowledge,* *please* *come to the* *group nutrition* *education class* *in 2 weeks”*	Ability Self-efficacy Reason

^1)^ motivational interviewing

## DISCUSSION

This is the first study to focus on the development of a nutritional intervention program for South Korean adolescent athletes applying the 5 A’s protocol. This program adopted the 5 A’s behavioral change counseling model and applied MI and OARS principles to provide effective nutritional intervention and drive behavioral changes in adolescent athletes. The theoretical framework and the 5 A’s counseling protocol were designed to support sustainable healthy eating habits, enhance nutrient intake, and improve the overall athletic performance of participants. In addition, we demonstrated that counseling samples and MI strategies can be used as motivators in each step of the 5 A’s counseling process and we developed sports nutrition educational materials for adolescent athletes in conjunction with nutrition experts.

Despite the increasing interest of student-athletes in the principles of sports nutrition, as a means to improve their performance in high-level sporting competitions, there are barriers to their application in their daily lives as a result of their busy academic and training schedules^27^. Although many adolescent athletes consider nutrition important for their athletic performance, their diets have been reported to fail in meeting the sports nutrition recommendations^28^^,^^29^. In addition, athletes’ awareness of the importance of nutrient intake in athletic performance does not always translate into eating habits^27^. A previous study by Partida et al. (2018) reported that female adolescent athletes had better nutritional knowledge than their male counterparts; however, male athletes were more likely to meet their dietary requirements^5^. As dietary habits and behaviors established in adolescence may persist into adulthood^30^, nutritional education and intervention, including healthy dietary patterns and nutrient intake strategies for before/during/after training, are necessary to ensure that adolescent athletes continue to have good habits in adulthood. Unfortunately, coaches often lack the nutritional knowledge needed to provide guidance on general health and athletic performance, this means that trained nutritional experts should educate adolescent athletes on these considerations^31^. In addition, a previous study by Karpinski et al. (2012) showed that athletes did not have the opportunity to receive adequate nutritional education from experts, including sports or clinical dietitians^32^.

Nutritional education for adolescent athletes should be based on principles of sound nutrition, including guidelines for appropriate nutrition in training and sports performance as well as nutrition for proper growth and development during adolescence^19^. Nutritional education programs for athletes should also be designed to enhance exercise performance, improve nutrient intake, and increase nutritional knowledge^33^. In this study, the contents of group nutritional education programs included basic nutrition concepts, basic food skills, performance nutrition, and performance enhancement, and were developed by experts to make it easier for adolescent athletes to learn about sports nutrition concepts and apply them to their everyday lives. The goal of the group education in this study was to ensure that adolescent athletes maintained healthy eating habits and positive body image to achieve a healthy lifestyle and high athletic performance. However, as education alone cannot guarantee positive behavioral changes, it is necessary to encourage adolescent athletes to change their dietary behavior by improving their personal awareness and skills through customized counseling^34^. Although most adolescent athletes understand that optimal nutrition is essential in training programs^35^, the vast majority of them do not link nutritional knowledge with practical food choice behaviors^36^.

The 5 A’s model used in this study has gained popularity as a counseling system to aid the client’s behavioral change and proved its effectiveness in smoking, dietary, drinking, and weight loss behavioral changes^12^^,^^26^^,^^37^. The 5 A’s is an attractive model because, in addition to being rooted in behavioral change theories, including self-management support, readiness assessment, behavioral modification, and self-efficacy enhancement, it can be conducted even when time is limited^26^. The 5 A’s counseling model can be implemented in approximately 10-20 minutes^12^, which may be helpful for adolescent athletes who have busy academic and/or training schedules. Health experts recommend the 5 A’s guidelines for assessing, advising, agreeing to, assisting, and arranging behavioral change efforts as evidence-based counseling tools in health counseling^37^^,^^38^. In each 5 A’s counseling process in this study, MI^39^, was used to increase motivation and encourage dietary behavioral changes. MI is a counseling technique that helps to re-enforce individual motivation and commitment to specific goals by deriving and exploring particular reasons for the change^40^. As MI is already used for counseling on exercise, diet, weight loss, bulimia, and anorexia^39^, it can be applied to nutritional counseling for adolescent athletes.

In this study, the basic principles and opening strategies of MI, combined with the 5 A’s model, provided for effective counseling of adolescent athletes. The basic principles of MI, including expressing empathy, developing discrepancy, rolling with resistance, and supporting self-efficacy^14^, may open up the participants and motivate them to change their behavior. In addition, the OARS (open-ended questions, affirmation, reflection, and summaries)^40^ principles of MI counseling techniques can help athletes set and achieve nutritional goals for training and competition.

As there are relatively few studies using the 5 A’s protocol in athletic programs, this study’s protocol was developed using the plans developed in previous studies^12^^,^^13^^,^^26^^,^^41^^-^^44^ on nutrition management in obesity. Nutrition and weight management of athletes is becoming increasingly important, as food selection for training and competition can lead to success and failure in sports^45^. Therefore, it is important for nutritional experts to understand the dynamic energy balance needed for competitive sports and develop a practical, evidence-based dietary approach to enable young athletes to achieve their goals to help them perform optimally in their respective sport^46^. Here we assumed that the 5 A’s model used for obesity management could apply to nutritional intervention for adolescent athletes, because although athletes who participate in weight-sensitive (e.g., endurance athletes, ski jumping), weight-class (e.g., wrestling, judo), or aesthetically judged (e.g., gymnastics, figure skating) sports are already lean, they may want to lose additional body fat to gain a competitive edge^47^. Through the assess, advise, agree, assist, and arrange phases of the 5 A’s counseling protocol, adolescent athletes may be able to set and achieve nutritional and weight management goals that can improve their health and athletic performance. Improving their health includes avoiding severe energy restriction, monitoring protein intake, nutrient timing, and quality, adopting a low-energy-dense (ED) diet, timing of food intake around exercise and spreading meals/snacks throughout the day, while monitoring the intake of ED beverages^48^. As the program proceeded several stages of assessment were undertaken with a full course of 5 A's taking place at each consultation. This was done in the hopes of changing the negative dietary behaviors of the participating athletes and reinforcing positive behavior.

The protocols and programs developed in this study have several strengths. This is the first protocol that applies psychological methodology (the 5 A's model and MI) to nutritional intervention for adolescent athletes. This protocol will motivate adolescent athletes to set and achieve their nutritional and weight management goals, while promoting healthy dietary behavior and athletic performance. It may also be applied to adult athletes. Intensive nutritional intervention combined with group nutrition education and the 5 A’s counseling process will significantly increase the nutritional knowledge of adolescent athletes and connect this knowledge to actual eating behavior. In addition, the nutritional intervention protocol developed in this study is easily accessible and can be generalized and extended to athletes who practice a variety of sports.

Despite its strengths, this study also has some limitations. Using only the MEDLINE/PubMed/Google Scholar database may result in limited verifications of relevant studies. As only a few previous intervention studies have focused on athletes, this protocol may need to be applied to participants and then corrected or supplemented. To minimize modifications, the latest nutrition-related data for adolescent athletes have been reviewed during the development of this study’s intervention protocol. Customized 5 A’s counseling with motivational interviewing requires a well-trained nutrition expert who understands a variety of sports and their specific training requirements to accurately assess each athlete and then apply this protocol. This means that proper training is required prior to protocol testing.

This study developed a nutritional intervention protocol for adolescent athletes using the 5 A’s framework and MI. It provides novel insights into how theory- and evidence-based nutritional intervention programs can support sustainable healthy eating habits, enhance nutrient intake, and improve athletic performance in adolescent athletes. Our results may allow the development of effective methods for implementing evidence-based nutritional interventions for adolescent athletes in rigorous training and competition environments. This nutritional intervention protocol and program could help establish nutritional strategies for the sound nutrition and athletic performance of both adolescent and adult athletes.
